# The clinical and cost-effectiveness of elective primary total knee replacement with PAtellar Resurfacing compared to selective patellar resurfacing: a pragmatic multicentre randomised controlled Trial with blinding (PART) - statistical analysis plan

**DOI:** 10.1186/s13063-026-09807-z

**Published:** 2026-05-20

**Authors:** R. N. Evans, A. Judge, G. S. Matharu, M. J. Petrie, A. W. Blom, K. Pike

**Affiliations:** 1 Bristol Medical School, Bristol Trials Centre, University of Bristol, Bristol, UK; 2 National Institute for Health and care Research Bristol Biomedical Research Centre, University Hospitals Bristol and Weston NHS Foundation Trust and University of Bristol, Bristol, UK; 3 Musculoskeletal Research Unit, Bristol Medical School, University of Bristol, Bristol, UK; 4 Sheffield Teaching Hospitals NHS Foundation Trust, Sheffield, UK; 5 School of Biological Sciences, The University of Manchester, Manchester, UK

**Keywords:** Statistical analysis plan, Total knee replacement, Patellar resurfacing

## Abstract

**Background:**

The PAtellar Resurfacing compared to selective patellar resurfacing Trial (PART) is a randomised controlled trial (RCT) to evaluate the clinical and cost-effectiveness of selective patellar resurfacing (intraoperative decision by surgeon) compared to always patellar resurfacing in patients undergoing elective primary total knee replacement (TKR). This paper presents the PART statistical analysis plan, detailing the trial analysis and presentation plan. The proposed analysis follows recommendations from the International Council for Harmonisation guideline on Good Clinical Practice and “Guidelines for the Content of Statistical Analysis Plans in Clinical trials” and are in line with the Consolidated Standards of Reporting Trials.

**Methods/design:**

PART is a multi-centre, randomised, blinded, pragmatic parallel two-group superiority RCT aiming to recruit 990 patients undergoing primary TKR. The primary outcome is the Oxford Knee Score (OKS) 12 months after TKR surgery. The planned statistical analyses of the trial primary and secondary outcomes are described in detail, including approaches to deal with the following: missing data, multiple testing, violation of model assumptions, withdrawals from the trial, non-adherence with the treatment and other protocol deviations.

**Discussion:**

This manuscript prospectively describes, prior to the completion of data collection and database lock, the analyses to be undertaken for the PART trial to reduce the risk of reporting bias and data-driven analyses.

**Trial registration:**

The trial was registered as ISRCTN33276681 on 27 Feb 2023, before recruitment began.

**Supplementary Information:**

The online version contains supplementary material available at 10.1186/s13063-026-09807-z.

## Introduction

Total knee replacement (TKR) is a clinically and cost-effective surgical procedure for treating patients with severe knee arthritis [1]. In the United Kingdom (UK) over 100,000 primary TKRs are performed a year, costing the National Health Service (NHS) £550 million annually [2, 3]. During the TKR surgery, surgeons have the option to perform a further procedure known as patellar resurfacing. The decision as to whether or not to perform patellar resurfacing in TKR is controversial, with potential advantages and disadvantages of both approaches. There is evidence that always patellar resurfacing (compared with never resurfacing) results in lower revision rates within 10 years of primary TKR surgery and is cost-effective given fewer patients need additional surgery in the long-term [4, 5]. Therefore, the National Institute for Health and Care Excellence (NICE) guidance published in June 2020 recommends a strategy of always patellar resurfacing in TKR over never resurfacing [3]. An alternative option is “selective patellar resurfacing” in which the surgeon decides on a case-by-case basis whether or not to resurface the patella based on their experience and an interoperative patient-specific assessment. Selective patella resurfacing was considered by NICE; however, there is no high-quality evidence comparing selective with always patellar resurfacing [6].

The ongoing PART trial is a multi-centre, randomised, pragmatic, parallel two-group superiority RCT with blinding. Patients, their clinical care team (except for staff directly involved in the surgery) and researchers responsible for follow-up will not be informed of the allocation. The primary aim is to evaluate the clinical- and cost-effectiveness of selective resurfacing compared to always resurfacing the patellar in adult patients undergoing elective primary TKR for osteoarthritis (OA) at NHS hospitals in England. Specific objectives are as follows: (a) To estimate the difference between resurfacing groups in the mean Oxford Knee Score (OKS) at 1 year postoperatively; (b) To estimate the difference between groups with respect to a range of secondary outcomes, including other knee-related Patient Reported Outcome Measures (PROMs), health-related quality of life, complications, further surgery and resource use and cost-effectiveness (both at 1 year postoperatively and longer-term). Full details of the study background and design have been reported elsewhere [7].

The trial opened to recruitment in April 2025, and the final participant was recruited in December 2025. At the time of writing, the trial is in the follow-up phase, with follow-up due to complete in December 2026 and results expected in autumn 2027. This paper describes the statistical analysis plan (SAP) for the PART trial clinical effectiveness and safety analyses. Cost-effectiveness analyses are covered in a separate health economics analysis plan and are not described in this paper.

## Methods and trial design

Presentation of study analyses is expected in summer 2027, after all participants have completed 1-year of trial follow-up and data has been cleaned and analysed.

### Outcomes

The primary outcome is the OKS at 12 months after TKR surgery. Secondary outcomes are described in detail elsewhere but briefly include knee pain and function at 3, 6 and 12 months, health-related quality of life, patient satisfaction, complications, length of surgery, post-operative length of hospital stay and further surgery of the patella [7].

### Sample size

The original planned sample size when the trial commenced was 530 participants. This was calculated to provide 90% power to detect a minimal clinically important difference of at least 4 points in the OKS [8], with a 5% two-tailed significance under the following assumptions:

i) OKS standard deviation (SD) of 10
ii) Correlation of 0.5 between the pre-surgery and post-surgery OKS and 0.7 between repeated post-surgery OKSs (conservative estimates)
iii) Up to 5% may not undergo resurfacing in the always-resurfacing group and up to 40% will undergo resurfacing in the selective-resurfacing group
iv) 10% loss to follow-up

The sample size calculation was revisited in June 2024, after 243 participants had been randomised, following concerns raised by the Data Monitoring and Safety Committee (DMSC) that the proportion of participants undergoing resurfacing in the selective-resurfacing group was higher than anticipated above. The above parameter estimates were therefore updated as follows:

i) OKS SD 8 points (updated from accruing trial data and assumed to be similar to baseline SD; based on *n* = 242)
ii) Correlation of 0.5 between pre-surgery and post-surgery OKS and 0.7 between repeated post-surgery scores (as per original calculation)
iii) Up to 2.5% may not undergo resurfacing in the always resurfacing group and that up to 65% will undergo resurfacing in the selective resurfacing group (updated from accruing data)
iv) 10% loss to follow-up (as per original calculation).

Recalculating the sample size to detect a target difference of 4 points in the OKS, with a 5% two-sided statistical significance level and 90% power resulted in a total revised sample size of 990 patients (495 per group). This was subsequently approved by the study’s DMSC, TSC and funder (approved in June 2025).

### Flow of participants

**Fig. 1:**
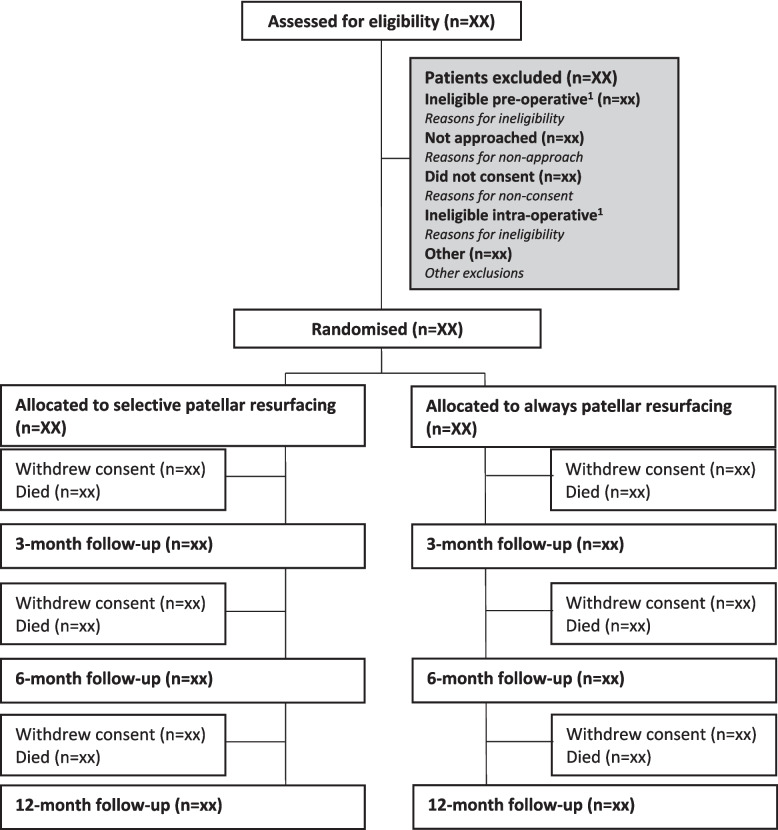
Flow of participants. Notes:^1^Patients may be ineligible for more than* one reason*

Participant flow through the study is described using a flowchart as recommended by the Consolidated Standards of Reporting Trials (CONSORT) [9] (Fig. 1). Screened patients fulfilling all eligibility criteria (described elsewhere) and giving written informed consent are randomised intraoperatively, once patient eligibility is confirmed by the surgeon [7]. The allocation is computer-generated and stratified by centre and implant type (cruciate retaining or cruciate sacrificing/posterior stabilised, which is a factor that can influence the decision to perform patellar resurfacing), according to permuted block randomisation, with blocks of varying size.

### Withdrawals/changes in participation status

Any participants who change participation status (e.g. withdrawn from treatment and/or follow-up at any stage of the study at their own discretion or that of the treating clinician) will continue to be followed up, unless they withdraw consent for further follow-up data to be collected. All data previously collected for the participant will be used in the analyses. Changes in participation status will be described by treatment allocation.

### Adherence with study protocol

Non-adherence with the trial protocol (randomisation of ineligible participants, failure to receive the allocated treatment and any notable breaches of good clinical practice) will be reported. The frequency of each type of protocol non-adherence will be described by treatment allocation.

### Analysis population

All summaries and analyses will be conducted on the intention-to-treat (ITT) population. The analysis (or ITT) population will consist of all participants randomised, according to their allocated intervention, regardless of whether they were found to be ineligible, prematurely discontinued treatment or were otherwise protocol deviators. A per protocol analysis will not be performed as the proportion of crossovers is expected to be low and can only occur in one randomised group, i.e. if randomised to always resurfacing the surgeon did not resurface. Therefore, we did not consider a per protocol analysis to provide meaningful additional information beyond the ITT analysis.

### Estimands

The estimand framework has been implemented to describe the treatment effect that PART sets out to quantify [10]. The primary aim is to evaluate the effect of selective resurfacing of the patellar during primary TKR surgery compared to always resurfacing the patellar if implemented as part of real-life routine clinical practice. Thus, the choice of the estimand attributes and strategies to handle intercurrent events have been chosen to reflect real-life practice, as described in Table 1.

**Table 1:** Estimand

Estimand component	Definition
Population	Adults listed for elective primary TKR for osteoarthritis at secondary and tertiary care NHS hospitals in England (as defined by trial inclusion/exclusion criteria)
Treatment condition	Selective resurfacing compared to always resurfacing
Endpoint	OKS at 1 year postoperatively
Summary measure	Difference in mean changes between treatment conditions
Handling of intercurrent events	The possible intercurrent events identified for this trial are: a) Patients who are randomised to always resurface but patella not resurfaced (treatment policy strategy—analysed according to randomised allocation) b) Patients who die during follow-up (hypothetical strategy—data included in model up to time of death) There are no remaining intercurrent events anticipated at this time

### Statistical analysis principles

Analysis principles and data presentation will follow recommendations from the International Council for Harmonisation guideline on Good Clinical Practice and are in line with the guidance issued in the CONSORT statement [9] and “Guidelines for the Content of Statistical Analysis Plans in Clinical trials” [11]; the corresponding checklist for the latter can be found in Supplementary Table 1. All statistical analyses will be carried out using the most recent version of Stata (statistical software) [12] at the time of analysis and will be performed after completion of all patient follow-up and database lock.

Where interventions are formally compared using statistical modelling, the always resurface group will act as the reference category. All applicable statistical tests will be two-sided and performed using a 5% significance level, with the exception of tests for interactions that will be performed using a 10% significance level. Confidence intervals (CIs) will be 95% unless otherwise stated.

No formal adjustment will be made for multiple testing; however, consideration will be taken in interpretation of results to reflect the number of statistical tests performed and the consistency, magnitude and direction of treatment estimates for different outcomes and analyses. Likelihood ratio tests will be used in hypothesis testing in preference to Wald tests.

### Descriptive analyses

Participant characteristics at randomisation (e.g. demography and baseline data, see Supplementary Table 2) and intra-operative details will be described by treatment allocation for the analysis population. Categorical data will be summarised using numbers and percentages, with continuous data summarised using the mean and SD (or median and interquartile range (IQR) if the data distribution is skewed). Any imbalances in the characteristics of the patients at the start of the study will be described; however, statistical tests for baseline imbalance will not be carried out.

### Outcome data analyses

All outcomes listed in the study protocol will be analysed under the umbrella of one of six types of outcome: (a) continuous outcomes measured at multiple time points, (b) continuous outcomes measured at a single time point, (c) binary, (d) time to event, (e) discrete and (f) descriptive. Table 2 classifies each study outcome.

**Table 2:** Data type of primary and secondary outcomes

Data type	Outcomes
Continuous at multiple time points	OKS OKS function subscale OKS pain subscale KOOS (pain, symptoms, function in activities of daily living, function in sport and recreation, and knee-related quality of life subscales) Health related QoL: EQ-5D (utility score and visual analogue scale [VAS] score)
Continuous at a single time point	Length of surgery (minutes)
Binary	Any complication Further surgery of the patella
Time to event	Length of post operative hospital stay (days)
Discrete (Likert scale)	Satisfaction with surgery: - Results of surgery - Results of your surgery for improving your pain - Results of your surgery for improving your ability to do home or yard work - Results of your surgery for improving your ability to do recreational activities
Descriptive	WOMAC (pain, stiffness and function subscales)
	Bang Blinding Index

Each outcome data type will be summarised and compared between the TKR resurfacing groups according to the following:

- *Continuous outcomes measures at multiple time points* will be summarised as means and SDs (or medians and interquartile ranges [IQRs] if distributions are skewed) in each resurfacing treatment allocation at each time point. Outcomes will be compared between groups using linear mixed effects methodology with treatment allocation and study design variables fitted as per section “Adjustment in models” below, and participant terms fitted as random effects. A time × treatment interaction will be fitted to allow changes in treatment effect with time to be described; the interaction will be included regardless of statistical significance to enable treatment effects at 3, 6 and 12 months to be estimated. Different variance/covariance structures will be explored, and the structure that provides the best fit in terms of information criteria such as Akaike’s Information Criteria (AIC), Bayesian Information Criteria (BIC) and likelihood ratio tests will be used. Treatment comparisons will be presented as adjusted mean differences (MDs) and 95% CI (or geometric mean ratios [GMRs] and 95% CIs if analysed on the log scale to improve model fit).
- *Continuous outcomes measured at a single time point* will be summarised by mean and SD (or median and IQR if distributions are skewed) in each resurfacing treatment allocation. Outcomes will be compared using linear regression. For untransformed data, treatment comparisons will be presented as adjusted differences in means with 95% CIs, and for logarithmically transformed data as adjusted GMRs with 95% CIs.
- *Binary outcomes* will be presented as numbers and percentages of patients in each treatment allocation. Outcomes will be compared between resurfacing treatment allocations using generalised linear models (Poisson family with log link or binomial family with identity link) with treatment comparison estimates presented as adjusted risk ratios (RR) or risk differences (RD) with 95% CIs. Formal statistical comparisons of treatment effects will only be performed if more than ten patients in total experience the outcome (with at least one event in each treatment allocation).
- *Time to event outcomes* will be summarised by the median and IQR in each treatment allocation, estimated from Cox proportional hazards models. If the assumptions of the Cox proportional hazard model are violated based on visual assessment of Kaplan-Meier (KM) curves, log(− log) plots and testing of scaled Schoenfeld residuals, alternative methods, such as restricted mean survival time or parametric models will be used as appropriate. Treatment comparisons will be presented as hazard ratios (HRs) and 95% CIs.
- *Discrete (Likert scale) outcomes* will be presented as numbers and percentages in each category in each resurfacing treatment allocation. Outcomes will be compared using ordinal logistic regression. If the assumptions of ordinal logistic regression are not met (e.g. proportional odds), alternative methods will be sought. This includes re-categorising the outcome, e.g. very satisfied and somewhat satisfied vs. very dissatisfied and somewhat dissatisfied, and comparing using generalised linear mixed models (Poisson family with log link or binomial family with identity link). A time × treatment interaction will be fitted to allow changes in treatment effect with time to be described; the interaction will be included regardless of statistical significance to enable the treatment effect at 3, 6 and 12 months to be estimated. Treatment comparison estimates will be presented as odds ratios (OR) with 95% CIs for ordinal logistic regression models, or RR or RD with 95% CIs for generalised linear mixed models.

- *Descriptive outcomes* will be described using either the mean and SD or the median and IQR (if data are non-normal) for continuous outcomes, or numbers and percentages for binary or categorical outcomes, by resurfacing treatment allocation and overall.

### Adjustment in statistical models

The intention is to adjust all statistical models for TKR implant type (cruciate retaining or cruciate sacrificing/posterior stabilised) as a fixed effect and adjust for hospital centre as a random effect (randomisation stratification factors). Time to event models will adjust for centre using a shared frailty term, if possible; however, if this leads to convergence issues, models will be stratified by centre.

For continuous outcomes that are also measured at baseline, post-randomisation values will be modelled as outcomes, and baseline values modelled as fixed effect covariates.

### Model assumptions

For all models underlying assumptions will be checked using standard methods, e.g. residual plots and tests for proportional hazards. If assumptions are not valid, then alternative methods of analysis will be sought, as already described. If outlying observations are found which mean models do not fit the data adequately, such observations will be excluded from the main analyses and comments made in footnotes. Sensitivity analyses may be performed to examine the effect on the study conclusions of excluding outlying observations.

### Missing data

A thorough data cleaning process will be carried out, and attempts will be made to obtain any missing data by chasing until it is either received or confirmed as not available.

In all tables, missing data for continuous variables will be indicated by footnotes. For categorical variables, missing data will be highlighted by the use of observation counts. If the amount of missing data differs substantially between resurfacing treatment allocations, then potential reasons will be explored.

- *Missing predictors:* There will be no missing data for any of the randomisation factors (by design). All other potential predictors are pre-randomisation measurements of continuous longitudinal outcomes. If data are missing for fewer than 5% of participants, the missing values will be imputed with mean values for the cohort as a whole. If data are missing for 5% or more of participants, then multiple imputation will be considered.
- *Missing outcomes:* For outcomes measured at one time point, if the proportion of missing data is less than 5%, then complete case analysis will be performed (i.e. excluding cases with missing data). If the proportion of missing data is 5% or more, then multiple imputation methods will be considered in place of a complete case analysis. A general imputation model that uses an iterative procedure to generate imputed values will be used to generate multiple complete data sets (e.g. using Stata’s mi impute command) [13]. The model of interest will then be fitted to each of the complete data sets and effect estimates combined using Rubin’s rules [14]. The imputation model will include all analysis variables, and any additional variables that predict missingness or known prognostic factors. If appropriate, methods such as predictive mean matching will be used in order to ensure that imputed values lie within specific ranges. As longitudinal models use all the available data across all time points, there is negligible benefit in imputing missing data if the missing data can be assumed to be missing at random (MAR) [15]. The assumption of data MAR will be examined by comparing the variances of the continuous outcomes across the groups and the change in participation status/dropouts in each group; if similar, the data can be assumed to be MAR [16]. It is anticipated that outcomes are more likely to be missing if the participant has a “poor” outcome; however, it is not expected that missingness will be related to treatment allocation. If the MAR assumption does not hold and dropout/missingness is not balanced across groups, imputation methods which do not assume MAR will be considered [17].

### Sensitivity analyses

No restriction on the time window for each questionnaire time point was specified in the trial protocol. The distribution of time windows for each questionnaire time point will be examined by treatment allocation. It is anticipated that the distribution will be balanced across treatment allocations. If any evidence of differing distributions between the groups is found, sensitivity analyses modelling time as a continuous covariate or excluding outcome scores completed outside of sensible time windows will be performed.

### Subgroup analyses

No subgroup analyses are planned.

### Safety data

Post-operative adverse events (AEs) and serious adverse events (SAEs) will be summarised by treatment allocation. Summaries of the total number of reported AEs and number of participants reporting at least one AE will be presented by treatment allocation. All unanticipated events (i.e. those not listed in the study protocol) will be coded using the most recent version of Medical Dictionary for Regulatory Activities (MedDRA) at the time of analysis.

## Discussion and trial status

We prospectively present the approach that will be taken in the analysis of the PART RCT, in advance of any outcome data analysis. Version 1 of the SAP was signed off in October 2023 (in the first year of trial recruitment) and version 2 in July 2025. Major changes made between the two versions included the increase in sample size from 530 to 990 as described earlier. Both versions were signed off prior to database lock and any statistical analysis of study outcome data. The SAP was shared with both the DMSC and the TSC, although formal sign-off by the committees was not required.

Publishing the SAP increases transparency and aids in deeper understanding of the methods used within the study. This transparency ensures results are not data- or method-driven and are not selectively reported.

## Supplementary Information

Additional file 1: Supplementary Table 1.

Additional file 2: Supplementary Table 2.

## Data Availability

Not applicable.

## Abbreviations

AE: Adverse event
CI: Confidence interval
CONSORT: Consolidated Standards of Reporting Trials
CTU: Clinical Trials Unit
EME: Efficacy and Mechanism Evaluation
EQ-5D-5L: EuroQol 5-Dimension
ISRCTN: International Standard Randomised Clinical Trial Number
MAR: Missing at random
MedDRA: Medical Dictionary for Regulatory Activities
MRC: Medical Research Council
NHS: National Health Service
NIHR: National Institute for Health and Care Research
OKS: Oxford Knee Score
REC: Research Ethics Committee
SAE: Serious adverse event
SAP: Statistical analysis plan
SD: Standard deviation
TKR: Total knee replacement
UK: United Kingdom
