# Supplementary material for: The clinical and cost-effectiveness of elective primary total knee replacement with PAtellar Resurfacing compared to selective patellar resurfacing: a pragmatic multicentre randomised controlled Trial with blinding (PART) - statistical analysis plan
Source: Trials. 2026 May 20;27:496. doi: 10.1186/s13063-026-09807-z (PMC13366729; doi:10.1186/s13063-026-09807-z)
Supplement: Supplementary file 1 — Additional file 1: Supplementary Table 1. [file 13063_2026_9807_MOESM1_ESM.pdf]

Statistical Analysis Plan Checklist

Gamble C, Krishan A, Stocken D, et al. Guidelines for the Content of Statistical Analysis Plans in Clinical Trials. JAMA. *2017;318(23):2337–2343. doi:10.1001/jama.2017.18556*

Study Name: PART

Recommended items to address in a Clinical trials SAP:

SECTION	Index	Description	Included	Reported on page #
**Section 1: Administrative Information**				
Title and trial registration	1a	Descriptive title that matches the protocol, with SAP either as a forerunner or subtitle, and trial acronym (if applicable)	☑	1
	1b	Trial registration number	☑	Header
SAP version	2	SAP version number with dates	☑	Footer
Protocol version	3	Reference to version of protocol being used	☑	4
SAP revisions	4a	SAP revision history	☑	23
	4b	Justification for each SAP revision	☑	23
	4c	Timing of SAP revisions in relation to interim analyses, etc	☑	N/A
Roles and responsibility	5	Names, affiliations, and roles of SAP contributors	☑	1, 4
Signatures of:	6a	Person writing the SAP	☑	4
	6b	Senior statistician responsible	☑	4

SECTION	Index	Description	Included	Reported on page #
	6c	Chief investigator/clinical lead	☑	1
**Section 2: Introduction**				
Background and rationale	7	Synopsis of trial background and rationale including a brief description of research question and brief justification for undertaking the trial	☑	4-5
Objectives	8	Description of specific objectives or hypotheses	☑	5
**Section 3: Study Methods**				
Trial design	9	Brief description of trial design including type of trial (e.g. parallel group, multi-arm, crossover, factorial) and allocation ratio and may include brief descriptions of interventions	☑	5
Randomisation	10	Randomisation details, e.g. whether any minimization or stratification occurred (including stratification factors used or the location of that information if it is not help within the SAP)	☑	5
Sample size	11	Full sample size calculation or reference to sample size calculation in protocol (instead of replication in SAP)	☑	6
Framework	12	Superiority, equivalence, or noninferiority hypothesis testing framework, including which comparisons will be presented on this basis	☑	6
Statistical interim analysis	13a	Information on interim analyses specifying what interim analyses will be carried out and listing of time points	☒	N/A
	13b	Any planned adjustment of the significance level due to interim analysis	☒	N/A
	13c	Details on guidelines for stopping the trial early	☒	N/A
Timing of final analysis	14	Timing of final analysis, e.g. all outcomes analysed collectively or timing stratified by planned length of follow-up	☑	4

SECTION	Index	Description	Included	Reported on page #
Timing of outcome assessments	15	Time points at which the outcomes are measured including visit “windows”	☑	7
**Section 4: Statistical Principles**				
Confidence intervals and p-values	16	Level of statistical significance	☑	9
	17	Description and rationale for any adjustment for multiplicity and, if so, detailing how the type ! error is to be controlled	☑	9
	18	Confidence intervals to be reported	☑	9
Adherence and protocol deviations	19a	Definition of adherence to the intervention and how this is assessed including extent of exposure	☑	8, 9
	19b	Description of how adherence to the intervention will be presented	☑	8
	19c	Definition of protocol deviations for the trial	☑	8
	19d	Description of which protocol deviations will be summarized	☑	8
Analysis populations	20	Definition of analysis population, e.g. intention to treat, per protocol, complete case, safety	☑	8, 9
**Section 5: Trial Population**				
Screening data	21	Reporting of screening data (if collected) to describe representativeness of trial sample	☒	N/A
Eligibility	22	Summary of eligibility criteria	☑	6,7
Recruitment	23	Information to be included in the CONSORT flow diagram	☑	17, 18
Withdrawal/follow-up	24a	Level of withdrawal, e.g. from intervention and/or from follow-up	☑	8

SECTION	Index	Description	Included	Reported on page #
	24b	Timing of withdrawal/lost to follow-up data	☑	8
	24c	Reasons and details of how withdrawals/lost to follow-up data will be presented	☑	18
Baseline patient characteristics	25a	List of baseline characteristics to be summarized	☑	28-32
	25b	Detailed of how baseline characteristics will be descriptively summarized	☑	18
**Section 6: Analyses**				
Outcome definitions		List and describe each primary and secondary outcome including details of:	☑	9-15
	26a	Specification of outcomes and timings. If applicable include the order of importance of primary or key secondary end points (e.g. order in which they will be tested.	☑	9-15
	26b	Specific measurement and units	☑	9-15
Analysis methods	27a	What analysis method will be used and how the treatment effects will be presented	☑	15-17
	27b	Any adjustment for covariates	☑	15
	27c	Methods used for assumptions to be checked for statistical methods	☑	16, 17
	27d	Details of alternative methods to be used if distributional assumptions do not hold e.g. normality, proportional hazards etc.	☑	16, 17
	27e	Any planned sensitivity analyses for each outcome where applicable	☑	18
	27f	Any planned subgroup analyses for each outcome including how subgroups are defined	☑	18
Missing data	28	Reporting and assumptions/statistical methods to handle missing data (e.g. multiple imputation)	☑	19

SECTION	Index	Description	Included	Reported on page #
Additional analyses	29	Details of any additional statistical analyses required e.g. complier average causal effect analysis	☒	N/A
Harms	30	Sufficient detail on summarizing safety data e.g. information on severity, expectedness, and causality; details of how adverse events are coded or categorized; how adverse event data will be analyses, i.e. grade ¾ only, incidence case analysis, intervention emergent analysis	☑	18
Statistical software	31	Details of statistical packages to be used to carry out analyses	☑	9
References	32a	References to be provided for nonstandard statistical methods	☒	N/A
	32b	Reference to data management plan	☒	N/A
	32c	Reference to the trial master file	☑	4
	32d	Reference to other standard operating procedures or documents to be adhered to	☑	4

Items that are important but do not necessarily need to be included in the SAP:

Index	Description	Included	Reported on page #
1	Summary of data cleaning carried out by the Statistician	☐	
2	Clear descriptions of how data cleaning will be performed and by whom	☐	
3	Summary of any complex checks carried out (e.g. checks conducted by the statistician as part of data cleaning)	☐	
4	Clear descriptions of specific complex checks to be performed and by whom (e.g. check missing randomisation numbers, check randomisation numbers are in chronological and sequential order)	☐	
5	Details of the location of the data to be analysed	☐	
6	Specification of how data will be merged and the unique parameters used for merging	☐	
7	List of any data importing and exporting to be carried out	☐	
8	Clear descriptions of how data importing and exporting will be done	☐	
9	Details of where data imported and exported will be stored	☐	
10	List and description of each primary and secondary outcome including details of any graphical representation of results	☐	
11	Dummy tables of all planned analyses (descriptive or comparative) presented in table format	☐	
12	Methods for handling outliers	☐	
13	Details of what data will be independently validated and quality checked e.g. source data verification	☐	
14	Details of how data validation and quality checking will be carried out	☐	

Index	Description	Included	Reported on page #
15	Method of how results will be validated (e.g. primary outcome, safety data and any unexpected results) and whether there will be independent programming of the primary outcome	☐	
16	Details of how final analysis datasets, programs and outputs will be archived at the end of the study analysis	☐	
17	Details of how final analysis datasets, programs and outputs will be shared with investigators at the end of the study analysis	☐	
