# Supplementary material for: The clinical and cost-effectiveness of elective primary total knee replacement with PAtellar Resurfacing compared to selective patellar resurfacing: a pragmatic multicentre randomised controlled Trial with blinding (PART) - statistical analysis plan
Source: Trials. 2026 May 20;27:496. doi: 10.1186/s13063-026-09807-z (PMC13366729; doi:10.1186/s13063-026-09807-z)
Supplement: Supplementary file 2 — Additional file 2: Supplementary Table 2. [file 13063_2026_9807_MOESM2_ESM.pdf]

Supplementary Table 2 Baseline demographics to be summarised

Baseline characteristic
Sex
Age
BMI
ASA score
Ethnicity
Employment
Smoking status
E-smoking status
Previous heart attack
Heart failure
Previous operation to unclog/bypass arteries in legs
Stoke/CVA/blood clot or bleeding in brain/TIA
Asthma
Emphysema, chronic bronchitis or COPD
Stomach ulcers or peptic ulcer disease
Diabetes
Poor kidney function
Received dialysis
Received kidney transplantation
Rheumatoid arthritis
Lupus
Polymyalgia rheumatica
Dementia
Cirrhosis or serious liver damage
Leukaemia or polycythaemia
Lymphoma
AIDS
Cancer, other than skin cancer, leukaemia or lymphoma
